# Solitary Polypoid Laryngeal Xanthoma

**DOI:** 10.1155/2013/967536

**Published:** 2013-05-20

**Authors:** Francisco Vera-Sempere, Diego Collado-Martín, Beatriz Vera-Sirera

**Affiliations:** ^1^Department of Pathology, La Fe University Hospital, School of Medicine and Dentistry, University of Valencia, Avenida Campanar 21, 46009 Valencia, Spain; ^2^Department of Otolaryngology, La Fe University Hospital, Avenida Campanar 21, 46009 Valencia, Spain; ^3^Department of Stomatology, School of Medicine and Dentistry, University of Valencia, Avenida Blasco Ibañez 17, 46010 Valencia, Spain

## Abstract

We report the case of a 51-year-old male smoker with diabetes mellitus and hyperlipidaemia and a long history of human immunodeficiency virus (HIV)/hepatitis C virus (HCV) infection treated with various antiretroviral regimes, who was referred to the otolaryngology department with progressive dysphonia. Fibre-optic laryngoscopy showed a solitary, yellowish-white pedunculated polyp on the anterior third of the left cord, with no other abnormality. Pathological analysis revealed a polypoid laryngeal xanthoma that was immunoreactive against CD68, perilipin, and adipophilin. This unusual laryngeal lesion in the clinical context of our patient suggests a possible role of antiretroviral treatment in the pathogenesis of these xanthomas.

## 1. Introduction

An xanthoma is a localised collection of fat-laden histiocytes that is not considered a true tumour but rather a reactive histiocytic proliferation [1]. They are often, but not always, a consequence of hyperlipidaemia and are sometimes indicative of specific lipoprotein disorders. The clinical presentation of xanthomas is variable, often affecting the skin and subcutaneous or tendinous tissues.

Mucosal xanthomas are uncommon [2] and a solitary xanthoma affecting the larynx is very rare; only two case reports have been published [3, 4]. We report a case of a solitary polypoid xanthoma of the vocal cord affecting a 51-year-old male smoker who had a long history of antiretroviral treatment for human immunodeficiency virus (HIV)/hepatitis C virus (HCV) coinfection, who presented with progressive dysphonia, and discuss the optical and immunohistochemical findings of this unusual lesion.

## 2. Case Report

A 51-year-old male smoker (1 pack/day for 35 years) was referred to the otolaryngology department with progressive dysphonia of 3-week duration. The dysphonia was intermittent initially and later became almost constant.

The medical history included an HIV-1 infection, diagnosed 25 years earlier and controlled with various antiretroviral drugs in the infectious diseases unit, with an acceptable current immunovirological status (CD4 529 cells/*μ*L and serum viral load undetectable). The patient had also been diagnosed with HCV coinfection (genotype 3a) and had been treated with interferon-ribavirin. Currently the patient was a diabetic on insulin, had a serum dyslipidaemia attributed to antiretroviral treatment, and was receiving atorvastatin (40 mg/day). The lipid profile showed the following: total cholesterol: 253 mg/dL, high-density lipoprotein (HDL): 103 mg/dL, low-density lipoprotein (LDL): 135 mg/dL, very low-density lipoprotein (VLDL): 15 mg/dL, and triglycerides: 76 mg/dL.

Fibre-optic laryngoscopy showed a solitary, yellowish-white, pedunculated polyp on the anterior third of the left cord, with no abnormalities elsewhere in the larynx. The lesion was easily resected from adjacent tissue via endolaryngeal microsurgery.

**Figure 1:**
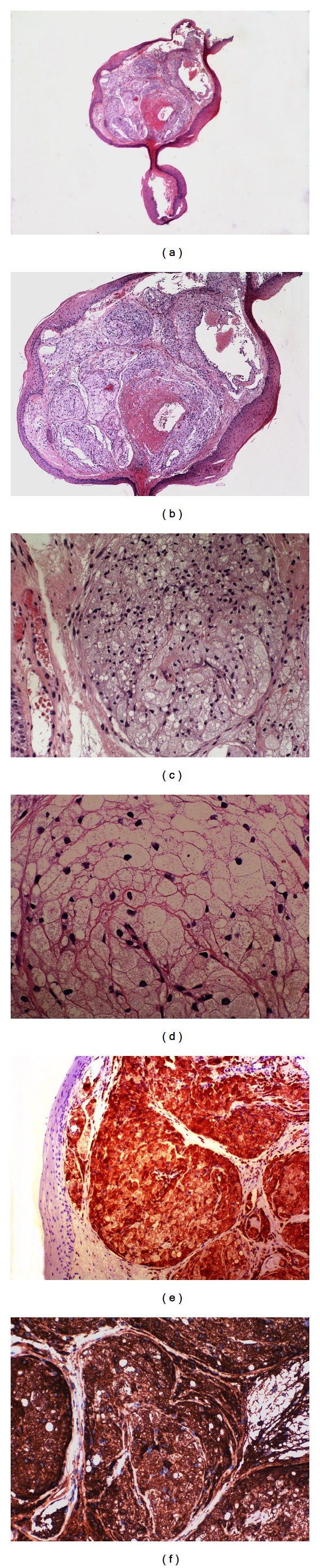
Panoramic histological view of the laryngeal polypoid lesion (1a) (H-E, 15x). Frequent islets of clear foam cells with congestive vessels are seen below a laryngeal squamous epithelium without atypia (1b) (H-E, 100x). At higher magnifications, the foam cells have a xanthomatous appearance (1c) (H-E, 250x) and are negative for PAS (1d) (PAS, 400x). Immunohistochemical staining shows intense cytoplasmic reactivity in the foam cells against CD68 (ie) (CD68, 250x) and adipophilin (1f) (adipophilin, 250x).

Histological examination of the resected specimen showed a laryngeal polyp that was composed internally of medium-to-large islets of foamy macrophages under a squamous epithelium, with small nuclei and without atypia, showing a degree of only superficial orthokeratinisation. There was no inflammatory component within the polyp but abundant vascularisation interspersed with islets of foam cells. The foam cells were negative for periodic acid-Schiff (PAS) and showed no granular character (Figures 1(a)–1(d)).

Immunohistochemical staining was performed on formalin-fixed, paraffin-embedded sections using the antibodies listed in Table 1. The foam cells were strongly positive for CD68 (Figure 1(e)) but not for cytokeratin AE1-3, S-100 protein, or CD1a. In addition, immunostaining with antiperilipin and antiadipophilin (Figure 1(f)) showed intracytoplasmic lipid inside the foamy cells, with membranous uptake at the periphery of lipid vacuoles, confirming the xanthomatous character of this laryngeal lesion.

## 3. Discussion

An xanthoma is defined as an aggregate of lipid-laden histiocytes. They are generally present in the skin and subcutis but can occasionally involve deep soft tissues (tendons or synovium) [1] and rarely bone, mucous membranes, or internal viscera [1, 2]. They are usually associated with a lipid abnormality, becoming an important clinical manifestation of disordered lipid metabolism, although there are poor correlations between circulating lipid levels and the corresponding size, number, or clinical relevance of xanthomas. Occasionally, xanthomas occur in normolipidaemic subjects and are thought to be the result of local cell dysfunction affecting lipid metabolism [1] and sometimes a cutaneous sign of internal malignancy or haematological dyscrasia [5], especially multiple myeloma.

Cutaneous xanthomas are classified, according to their gross appearance and clinical presentation, as eruptive, planar, tuberous, or tendinous [1]. The typical histological pattern of an xanthoma is a well-circumscribed solid nodular collection of foamy histiocytes, with bland nuclei, small inconspicuous nucleoli, and abundant vacuolated cytoplasm. Sometimes, particularly in the tuberous and tendinous forms, extracellular cholesterol deposits, fibrosis, and associated inflammation are seen, with this last component also present in the eruptive forms [1].

Noncutaneous xanthomas are infrequent and exceptional in the larynx, with only eight cases reported [6]. Some of these cases involve disseminated xanthomas in the larynx [7]. Our patient had no other xanthomas on the skin or at any other location. A solitary xanthoma of the larynx is very rare, with only two reported [2, 3], interestingly both in non-HIV-infected, normolipidaemic patients.

Cutaneous xanthomas usually present few problems in diagnosis and management. Given their nonneoplastic nature, conservative, medical therapy is generally recommended [1]. Noncutaneous xanthomas might be more problematic to diagnose. In the case of a laryngeal xanthoma, the differential diagnosis includes several processes, including xanthoma disseminatum affecting the larynx [7], which we ruled out clinically because of the absence of other xanthomas in the skin. Histologically, we also excluded the diagnoses of verruciform xanthoma and granular cell tumour of the larynx because of the morphological appearance of nonpapillary squamous epithelium observed on the surface and the absence of PAS- and S-100-positive granulations. In addition, with perilipin and adipophilin, two recently described antibodies that recognise proteins associated with lipid vesicles in formalin-fixed paraffin-embedded tissue [8], we demonstrated unequivocally the presence of lipid droplets inside foam cells and therefore the xanthomatous character of the lesion. Another possibility that had to be excluded in our case is the so-called papular neutrophilic xanthomas described in HIV-positive patients. The absence of neutrophils, leukocytoclasis, and nuclear debris at a histological level excluded this entity, considered by some as distinctive of HIV infection and described in patients with an underlying lymphoproliferative disease [9].

The clinical context of our patient, with a long history of treatment with various antiretroviral regimes, provides a hypothesis of the pathogenesis of the polypoid laryngeal xanthoma. At the time of diagnosis, the patient was a diabetic on insulin and had marked hyperlipidaemia, which were attributed to long-term antiretroviral combination therapy [10]. Active antiretroviral therapy in HIV-infected patients leads to significant extension of life expectancy, but these benefits are tempered by a broad spectrum of side effects [10], including a wide range of laboratory (hyperlipidaemia and insulin resistance) and clinical (mainly increased cardiovascular risk) disturbances. The occurrence of xanthomas in unusual locations, as seen in our patient, could be added to this list. Larger studies with long-term followup are required to determine the risk of xanthoma formation associated with prolonged antiretroviral therapy.

## Figures and Tables

**Table 1:** Antibodies used in the immunohistochemical study.

Antibody specificity	Clone	Dilution	Source
AE-1-3	AE1/AE3	1/50	Dakopatts, Glostrup, DK
P-S-100	Polyclonal	RtU	Dakopatts, Glostrup, DK
CD68	KP1	RtU	Dakopatts, Glostrup, DK
CD1a	010	1/50	Dakopatts, Glostrup, DK
Perilipin	PERI 112.17	RtU	PROGEN Biotechnik, Heidelberg, DE
Adipophilin	AP 125	RtU	PROGEN Biotechnik, Heidelberg, DE

RtU: ready to use with Envision Flex+.
